# CircMYO9A inhibits influenza A virus replication by dampening haemagglutinin cleavage via increasing SERPINE1/PAI-1 expression

**DOI:** 10.1080/22221751.2025.2502007

**Published:** 2025-05-02

**Authors:** Yiqing Zheng, Xiaoting Zhang, Zhiyuan Liu, Menglu Fan, Lulu Deng, Jihui Ping

**Affiliations:** MOE Joint International Research Laboratory of Animal Health and Food Safety, Engineering Center of Animal Immunity of Jiangsu Province, College of Veterinary Medicine, Nanjing Agricultural University, Nanjing, People’s Republic of China

**Keywords:** Influenza virus, circMYO9A, SERPINE1, CeRNA, haemagglutinin cleavage

## Abstract

Circular RNAs (circRNAs) represent a class of widespread and diverse covalently closed circular endogenous RNAs that play critical roles in regulating gene expression in mammals. However, the roles and regulatory mechanisms of circRNAs during influenza A virus (IAV) infection remain largely unexplored. In this study, we screened the circRNA transcription profiles of WSN-infected cells to identify circRNAs involved in viral replication and identified a novel differentially expressed circular RNA, circMYO9A. Mechanistically, circMYO9A acts as a competing endogenous RNA (ceRNA) for SERPINE1/PAI-1 by sponging miR-6059-3p, thereby increasing SERPINE1/PAI-1 expression, which restricts IAV haemagglutinin cleavage and subsequently reduces the infectivity of progeny viruses. Importantly, our findings demonstrate that circMYO9A significantly inhibits viral replication in the lungs of infected mice, potentially increasing their survival during IAV infection. These results demonstrate that circRNAs play crucial roles in inhibiting IAV replication and provide novel insights into potential therapeutic strategies involving circRNAs.

## Introduction

Circular RNAs (circRNAs), an emerging family of RNAs, originate from nonsequential back-splicing of exons and/or introns of precursor messenger RNAs (pre-mRNAs) [1,2]. Owing to the absence of RNA ends, circRNAs exhibit resistance to cellular exoribonucleases and are thus extremely stable. Previously, circRNAs were thought to be byproducts of unusual splicing with no function or minute functional activity. However, owing to rapid progress in the fields of high-throughput RNA sequencing (RNA-seq) and circRNA-specific computational biology, a wide array of circRNAs have been reported in eukaryotes, and their functions are being explored [3]. Although circRNAs are single-stranded RNAs, they vary from linear RNAs because they are closed covalently, which endows circRNAs with fascinating properties, such as parental gene modulation, protein complex scaffolding, microRNA (miRNA) or protein sponging, and RNA-protein interactions [4]. Recent studies have shown that circRNAs participate in many physiological and pathological processes by regulating epigenetic modifications, gene transcription, alternative splicing, RNA stability, and protein translation and serve as new diagnostic and treatment strategies for disease [5–7]. Nevertheless, compared with coding RNAs, miRNAs and long noncoding RNAs (lncRNAs), there are still significant gaps in our understanding of circRNAs. The biological and molecular mechanisms of circRNAs in the development of various diseases are not yet fully understood. Further studies will reveal the functions of the vast majority of circRNAs in terms of both physiological and pathological processes.

Analysis of RNA-seq data obtained from cells infected with diverse viruses revealed changes in host circRNAs in response to viral infection [8–10]. Increasing evidence suggests that circRNAs are involved in viral infection and progression through distinct mechanisms [11–13]. A study by Li and colleagues revealed that NF90/NF110 in the nucleus promotes circRNA biogenesis and is often found in the circRNP complex; however, upon viral infection, NF90/NF110 is released from circRNP complexes and binds to viral RNAs, thereby inhibiting virus replication [14]. miRNA sponging is one of the well-established functions of circRNAs, where they can inhibit the function of miRNAs by binding target miRNAs directly or indirectly. Multiple reports have revealed various differentially expressed host circRNAs and established a circRNA-miRNA-mRNA regulatory network involved in immune responses to infections with multiple viruses, including Zika virus, Seneca virus A, influenza A virus, and coxsackievirus B3 [15–18]. Owing to the absence of the 5’cap and the poly(A) tail, circRNAs are unable to undergo cap-dependent translation. However, increasing evidence has demonstrated that some circRNAs can encode peptides through cap-independent translation mechanisms, including internal ribosome entry sites (IRESs) and N6-methyladenosine (m6A) modifications, such as circMORC3 and circEZH2, which further expands our understanding of their roles in immune responses [19,20]. Increasing evidence indicates that circRNAs participate in innate and adaptive immunity. However, the underlying mechanisms of most circRNAs in virus infection remain largely unexplored. Analysing the relationship between circRNAs and viral infection will provide fresh insights into antiviral immunity, facilitating the development of potential therapeutics.

Influenza A viruses (IAVs), zoonotic pathogens that continuously circulate, pose serious burdens and threats to global public health [21–23]. IAV belongs to the family Orthomyxoviridae and contains the envelope glycoproteins haemagglutinin (HA) and neuraminidase (NA). The influenza virus haemagglutinin (HA) mediates the first essential step in the viral life cycle: virus entry into target cells [24]. The HA protein binds sialic acid-terminating surface receptors and actively induces membrane fusion within endosomes during the process of viral entry [25]. Influenza virus HA, which is synthesized as an uncleaved HA0 precursor in infected cells, is unable to cause membrane fusion and must first be cleaved by host cell proteases into a fusion-competent HA1/HA2 complex [26]. After virus budding and HA0 cleavage, an influenza virus is capable of infecting a new host cell. Analysis of RNA-seq data obtained from cells infected with IAV revealed that the expression levels of most circRNAs changed significantly after IAV infection [27–29], but the role of circRNAs in IAV infection remains largely unexplored. According to recent studies, circRNAs have the potential to affect IAV replication. The upregulation of the circRNA AIVR by IAV inhibits viral replication by sponging miRNAs and promoting the expression of CREBBP to facilitate IFN-β production [17]. circVAMP3 serves as a decoy for the viral NP and NS1 proteins, which interfere with vRNP complex activity and inhibit the effect of NS1 on the RIG-I signalling pathway [30]. However, the biogenesis processes, underlying functions, and potential mechanisms of circRNAs in IAV infection remain elusive.

In this study, we screened the circRNA transcription profiles of WSN-infected cells and identified dysregulated circRNAs closely related to the pathogenic process of influenza virus. We determined that circMYO9A, whose expression was greatest upon IAV infection, inhibited IAV replication and revealed the underlying mechanism by which circMYO9A exerts its antiviral effects. Mechanistically, circMYO9A inhibits viral replication by sponging miR-6059-3p as a competing endogenous RNA (ceRNA) for SERPINE1/PAI-1 and upregulating SERPINE1 expression, which restricts IAV haemagglutinin cleavage and subsequently reduces the maturation of progeny particles. Importantly, our findings demonstrate that circMYO9A significantly inhibits viral replication in a mouse model, potentially enhancing mouse survival during IAV infection. These results demonstrate that circRNAs play crucial roles in inhibiting IAV replication and provide novel insights into potential therapeutic strategies involving circRNAs.

## Materials and methods

### Viruses

Influenza viruses A/WSN/33 (H1N1) and A/Hong Kong/1/68(H3N2) were grown on MDCK cells. A/chicken/Anhui/LH99/2017(H9N2) was propagated in 10-day-old specific pathogen-free (SPF) embryonated chicken eggs. Viral titres were measured by plaque assay in MDCK cells.

### Cells

Adeno carcinomic human alveolar epithelial (A549) cells, Madin Darby Canine Kidney (MDCK) cells and Human embryonic kidney (HEK293T) cells were cultured in Dulbecco’s Modified Eagle’s medium (DMEM; Gibco, USA) containing 10% fetal bovine serum (FBS; Sbjbio, China), 0.2% NaHCO_3_, 100 μg/mL streptomycin, and 100 IU/mL penicillin (Gibco, USA) at 37°C with 5% CO_2_.

### Antibodies

anti-GAPDH (10494-1-AP; Proteintech); anti-NP antibodies (kindly provided by Dr. Chengjun Li, Harbin Veterinary Research Institute, The Chinese Academy of Agricultural Sciences); anti-FLAG (SLAB0102; Smartlife sciences, China); anti-HA antibodies generated by our lab as previous reported [31].

### Oligonucleotides

The sequences for primers and oligonucleotides used in this study are provided in Table S1. The primers for plasmid construction and qRT-PCR were synthesized by General Biol (Anhui, China) and Sangon Biotech (Shanghai, China). The probes used for FISH, siRNAs, miRNAs, and miRNA inhibitors were synthesized by GenePharma (Shanghai, China). The 5’ biotin-labelled tagged probes used for circRNA precipitation were synthesized by TsingKe (Beijing, China).

### Plasmid construction

The circRNA-overexpressing plasmid pcDNA-circMYO9A was constructed by using the pcDNA3.1 backbone as described previously [32] with minor modifications. Briefly, the intron 4 fragment of MLLT3/AF9 (chr9, 20414651 to 20415428; hg38) was inserted into EcoRI/EcoRV sites of pcDNA3.1. Subsequently, the full-length linear sequence of circMYO9A with a 135-nt upstream flanking sequence and a 131-nt downstream flanking sequence and the inverted fragment of the above intron 4 fragment of MLLT3/AF9 were overlapped and then were inserted into EcoRV/XhoI sites. The intron 4 fragment of MLLT3/AF9 (chr9, 20414651 to 20415428; hg38) was inserted to facilitate the circularization of the inserted circMYO9A linear sequence.

The circMYO9A-ORF-Flag and the SERPINE1-Flag expression vectors were constructed by inserting the coding region (915nt and 957nt) into PCAGGS-Flag vector. The AGO2-Flag expression vector was constructed by inserting the AGO2 coding region(2580nt) into PCMV-Flag. The SERPINE1 expression reporter vectors were constructed by inserting the SERPINE1 coding region with 3’UTR sequences (2400nt) into PCMV-Flag. The pmirGLO-circMYO9A and pmirGLO-SERPINE1-UTR vectors were constructed by inserting the full-length circMYO9A (911nt) or SERPINE1-UTR with the length of 1000nt containing the potential target sequences into pmirGLO vector, respectively. The pmirGLO-circMYO9A and pmirGLO-SERPINE1-UTR mutants were constructed by overlap PCR. All primers used to construct plasmids are listed in TableS1. All gene cloning was performed using Phanta Max Super-Fidelity DNA polymerase (Vazyme, China), and all constructs were verified by Sanger sequencing.

The circMYO9A sequence was cloned into pHBAAV-CMV-circRNA-EF1-ZsGreen by HANBIO Co., Ltd. to package AAV6-circMYO9A.

### Virus infection, RNA extraction, library construction, and sequencing

A549 cells were infected with WSN/33 virus at an MOI of 3 for 12 h, mock-infected cells were placed in the same volume of DMEM, with the same concentration of TPCK-treated trypsin. Total RNA was isolated from each group using RNA isolater Total RNA Extraction (Vazyme, China) according to the manufacturer’s instructions. circRNA and mRNA library construction: after total RNA was extracted, rRNAs were removed to retain mRNAs and ncRNAs. The enriched mRNAs and ncRNAs were fragmented into short fragments by using fragmentation buffer and reverse transcribed into cDNA with random primers. Next, the cDNA fragments were purified with QiaQuick PCR extraction kit (Qiagen, Venlo, The Netherlands), end repaired, poly(A) added, and ligated to Illumina sequencing adapters. Then UNG (Uracil-N-Glycosylase) was used to digest the second-strand cDNA. The digested products were size selected by agarose gel electrophoresis, PCR amplified, and sequenced using Illumina HiSeqTM 4000 by Gene Denovo Biotechnology Co. (Guangzhou, China).

Small RNA library sequencing: after total RNA was extracted, the RNA molecules in a size range of 18–30nt were enriched by polyacrylamide gel electrophoresis (PAGE). Then the 3’ adapters were added and the 36–48nt RNAs were enriched. The 5’ adapters were then ligated to the RNAs as well. The ligation products were reverse transcribed by PCR amplification and the 140–160 bp size PCR products were enriched to generate a cDNA library and sequenced using Illumina HiSeq Xten by Gene Denovo Biotechnology Co. (Guangzhou, China).

### RT–PCR and RT-qPCR

Extracted RNAs were used for RT–PCR or RT-qPCR. For relative quantitative analysis, cDNAs were synthesized using HiScript II Q RT Super Mix for qPCR with genomic DNA (gDNA) eraser (Vazyme, China). The real-time PCR assays were performed using AceQ qPCR SYBR green master mix (Vazyme, China) in a Roche Light Cycler 96, according to the manufacturer’s instructions using the following cycling programme: 95°C for 5 mins, 40 cycles at 95°C for 10 s, and 60°C for 30 s. The 2^^(-ΔΔCt)^ method was used to determine the relative levels of candidate genes. The glyceraldehyde-3-phosphate dehydrogenase (GAPDH) was used to normalize the relative expression levels of circular and linear RNAs. All RT–PCR and RT-qPCR primers used in this study are listed in Table S1.

### Immunofluorescence assay (IFA)

A549 cells in 6-well plates with 90% confluence were mock infected or infected with WSN at an MOI of 3 for 12 h. Cells were washed three times with phosphate-buffered saline (PBS) and fixed with 4% paraformaldehyde and permeabilized with a solution of PBS containing 0.2% Triton X-100 for 10 min (Sigma). After blocking in 5% skim milk for 1 h at room temperature, cells were incubated with mouse polyclonal antibody recognizing the IAV NP protein at 37°C for 1 h and subsequently incubated with a fluorescein isothiocyanate (FITC)-labelled goat anti-Mouse secondary antibody at 37°C for 1 h, followed by treatment with 4,6-Diamidino-2-phenylindole dihydrochloride (DAPI) at room temperature for another 10 min. Fluorescence images were captured using a Nikon TI-S inverted fluorescence microscope (Nikon, Japan).

### Cell transfection and infection

Transient transfection of cells with miRNA mimic, miRNA inhibitor, siRNA or DNA plasmids was performed in 12-well plates using Lipofectamine 3000 (Invitrogen) according to the manufacturer’s instructions. For functional analyses, the overexpression plasmid (1μg per well) or control vector (1μg per well) and miRNA mimics (80nM), miRNA inhibitor (80nM), or siRNA (80nM) were transfected into cells in culture medium. To investigate the effect of these transfections on the growth of influenza viruses, the above-transfected cells were infected with virus at the indicated MOI for the indicated hours in each experiment. Supernatants were collected, and virus titres were determined in MDCK cells. For luciferase experiments, was performed in 24-well plates, miRNA mimics (80nM) or miRNA inhibitor (80nM) and pmirGLO (100 ng per well) containing the wild or mutated plasmid were transfected into cells and then harvested for further detection.

### RNase R digestion

Total RNA (10 μg) was incubated for 30 min at 37°C with or without 3 U/μg of RNase R (Beyotime, China) and then Deactivate at 70℃ for 10 min. The resulting RNA was purified by a second phenol–chloroform extraction. Then, the treated RNAs were reverse transcribed and detected by RT–PCR assay with a divergent primer or convergent primer followed by nucleic acid electrophoresis.

### Plaque assay

The infectious titres of influenza viruses were determined by plaque assays according to the procedures described previously. Briefly, viruses were serially 10-fold diluted and inoculated onto MDCK cell monolayers. After incubation at 37°C for 60 min, the cells were overlaid with DMEM containing 1% Sea Plaque agarose (Lonza) and 1μg/mL TPCK-trypsin and incubated at 37°C. At 2 days post infection (d.p.i.), check for plaque formation.

### Subcellular fractionation

Nuclear and cytoplasmic RNA were isolated separately using the Cytoplasmic & Nuclear RNA purification kit (Norgen Biotek, Thorold, ON, USA) according to the manufacturer’s protocol.

### Western blotting

Proteins in the lysates were separated by SDS-PAGE, transferred to nitrocellulose membranes (catalog number 10600001; GE Amersham). The membrane was then blocked with 5% skim milk and incubated with primary antibodies at 4°C overnight. After hybridization with horseradish peroxidase (HRP)-conjugated secondary antibodies, the membrane was visualized using enhanced chemiluminescence (ECL) reagents (Vazyme, China), and the signals were analysed using an Amersham Imager 600 charge-coupled (device) CCD-based chemiluminescent analyzer (GE Healthcare).

### Fluorescence in situ hybridization (FISH)

FISH assays were performed using Fluorescence in Situ Hybridization Kit (Gene Pharma, Shanghai, China) according to the instructions. Briefly, A549 cells were seeded on glass coverslips and infected with WSN at MOI 3 or transfected with circMYO9A plasmid. The cells were fixed with 4% paraformaldehyde at 12 h after infection and then permeabilized with 0.1% Triton X-100. After washing with 1x SSC (Saline Sodium Citrate Buffer) /50% formamide, the cells were incubated with the probe overnight at 37°C. DAPI was used for nuclear staining. The following 5’ cy3-labelled FISH probe to the circRNA of were used: 5’-AAATAACTCAAGTACTGGTCCAGCT-3’ (synthesized by Genepharma, Shanghai, China).

### Dual luciferase reporter assay

HEK-293T cells were seeded in 24-well plates 24 h before transfection. Cells were co-transfected with a mixture of 100 ng vector (pmirGLO-circMYO9A or pmirGLO-empty vector) and miRNA mimics (80nM) using Lipofectamine 2000 (Invitrogen) according to the manufacturer’s instructions. Transfection of each construct was performed in triplicate in each assay. The miRNA mimics were synthesized by Gene Pharma (Shanghai, China). After 36 h after transfection, cells were lysed with 100μL of passive lysis buffer (Promega), and firefly and renilla luciferase bioluminescence was measured using a dual-luciferase system (Promega) according to the manufacturer’s instructions. The firefly luciferase activity was normalized with its corresponding renilla luciferase activity.

### RNA pulldown assay

Antisense Oligo Pulldown of Circular RNA: A biotin-labelled circMYO9A probe was designed to be complementary to the head-to-tail junction site. The negative-control biotin-labelled probe was a nonsense sequence. The ASO-RNA pulldown assay was conducted in A549 cells (∼2 × 10^^7^) transfected pcDNA3.1 and pcDNA-circMYO9A. After 48 h transfection, Cells were washed twice with cold PBS and then lysed by 1 mL of radioimmunoprecipitation assay (RIPA) lysis buffer (Beyotime, China) and 1uL of RNase inhibitor (Beyotime, China) for 30 min on ice. The lysates were precleared by centrifugation for 5 min, and 100μL of the sample was aliquoted for input. The remaining lysates were incubated with M-280 streptavidin magnetic beads (Sigma) conjugated with biotin-labelled probe at 4°C for 16 h. After the magnetic beads were washed 5 times with cold lysis buffer in a magnetic field, the coprecipitated RNA were extracted with TRIzol and evaluated by qPCR.

To conduct pulldown assay with biotinylated miRNA, A549 cells were harvested at 48 h after transfection, then incubated on ice for 30 min in RIPA lysis buffer with RNase inhibitor. The lysates were precleared by centrifugation for 5 min, and 100μL of the sample was aliquoted for input. The remaining lysates were incubated with M-280 streptavidin magnetic beads (Sigma) conjugated with biotin-labelled miRNA at 4°C for 16 h. After the magnetic beads were washed 5 times with cold lysis buffer in a magnetic field, the coprecipitated RNA and 10% input were extracted with TRIzol and evaluated by qPCR.

### RNA immunoprecipitation

The Ago-RIP assay was conducted in A549 cells (∼2 × 10^^7^) transfected pcmv-Ago2-flag or pcDNA3.1-empty vector and pcDNA-circMYO9A. After 48 h transfection, the cells were extract and incubated with magnetic beads conjugated with IgG and anti-Flag antibody (Smartlife sciences, China). RNA was extracted from the remaining beads and qPCR was used to evaluate the expression levels of circMYO9A.

### Animal modelling and in vivo infection

The mice were anesthetized with isoflurane, after which AAV6 expressing circMYO9A purchased from Hanheng Company (Hanheng Biotechnology, China) was intratracheally instilled into the mice (5 × 10^^10^ vg/mL per mouse). After 21 days, the mice were challenged with the WSN strain at 5 × 10^^3^ PFU per mouse by nasal drip. Body weights and survival were recorded up to 14 days post-infection (d p.i.). The lungs of the mice were harvested and placed in 10% buffered formalin for histological sectioning or 1 mL of PBS for detection of the viral titre. The fixed samples were then embedded in paraffin and used for immunofluorescent staining with the anti-NP mAb or haematoxylin and eosin (H&E) staining.

### Statistical analysis

The data are shown as mean values ± standard deviations, and unless otherwise indicated, all the data presented are representative results of at least three independent repeats. Statistical analysis was performed with Prism 8 (GraphPad), and the statistics were analysed by two-tailed Student’s t test or one-way or two-way analysis of variance (ANOVA) as indicated. Differences considered to be significant are indicated by asterisks as follows: *, *P *<* *0.05; **, *P *<* *0.01; ***, *P *<* *0.001; ****, *P *<* *0.0001. “ns” indicates no significance.

## Results

### Identification of differentially expressed circRNAs in WSN-infected A549 cells

**Figure 1.**
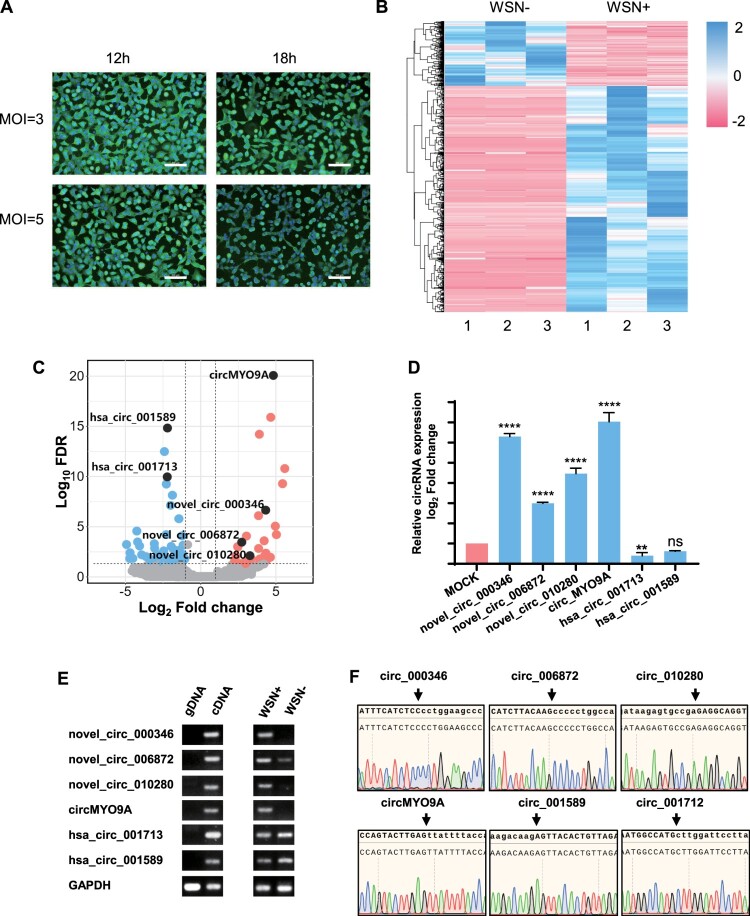
Identification of differentially expressed circRNAs in WSN-infected A549 cells. (A) IFA image of mock and WSN infected A549 cells. Cells were infected with or without WSN at different MOI and different time points and then stained with anti-IAV NP protein monoclonal antibody(green). Nucleic acids were labelled with 4’,6-diamidino-2-phenylindole (blue). Scale bar, 500 μM. (B) Heatmap of circRNA-seq analysis of mock- and WSN-infected A549 cells. RNA-Seq analysis revealed 784 upregulated and 224 downregulated circRNAs in WSN-infected A549 cells compared with WSN-uninfected A549 cells (*n* = 3; |fold change|>2; *P *<* *0.05) Relative expression values are indicated based on colour and decrease in value from blue to red (log scale 2, from +2 to −2). (C) Volcano plot constructed using fold change values and FDR values. Red dots represent upregulated circRNAs, and blue dots represent downregulated circRNAs. Black dots are candidate circRNAs. (D) Validation of the differential expression of circRNAs upon influenza virus infection. Expression levels of the six dysregulated circRNAs analysed by RT-qPCR in A549 cells with or without WSN infection. A549 cells were infected with or without WSN at an MOI of 3 for 12 h, and total RNA was isolated for RT-qPCR analysis. RT-qPCR data were normalized against GAPDH mRNA. Relative expression of circRNA was normalized against the mock infection group. Data shown are the mean ± SD of three samples. (E) RT-PCR validation for the six dysregulated circRNAs. The divergent primers were designed for cDNA and genomic DNA (gDNA) PCR amplification. (F) Sanger sequencing validation showing Junction sites of the six circRNAs.

To investigate the differential circRNA expression in WSN-infected cells, we infected A549 cells with WSN of different MOI. At MOI = 3 and 12 h after infection (hpi), RNA-seq was performed on both uninfected and WSN-infected A549 cells (Figure 1(A)), which could ensure the complete replication cycle of the virus and enable A549 cells to completely infected. The expression patterns of these dysregulated circRNAs are shown in Figure 1(B). A total of 1008 dysregulated circRNAs were identified in WSN-infected A549 cells, including 784 upregulated and 224 downregulated circRNAs. Among these, 574 (56.94%) were newly identified, whereas 434 (43.06%) were previously reported in circBase [33]. These circRNAs were identified through transcriptome sequencing and bioinformatics analysis. To minimize false-positive data, four upregulated and two downregulated circRNAs, comprising three known circRNAs from circBase and three newly identified circRNAs, were selected for further validation. These circRNAs were validated by RT-qPCR and RT–PCR (Figure 1(C, E right panel)). RT-qPCR confirmed that the expression patterns of these six circRNAs were consistent with the RNA-seq results. Notably, circMYO9A (hsa_circ_0006509) was the most significantly upregulated circRNA upon IAV infection. To further confirm the presence of these circRNAs, divergent primers were designed for cDNA and genomic DNA (gDNA) PCR amplification (Figure 1(E left panel)). Sanger sequencing validated the PCR product, confirming that the head-to-tail splice junction site was amplified by the divergent primers (Figure 1(F)). These findings suggest that endogenous circRNAs are likely involved in IAV infection and that many newly identified circRNAs warrant further exploration.

### Characteristics of circMYO9A

**Figure 2.**
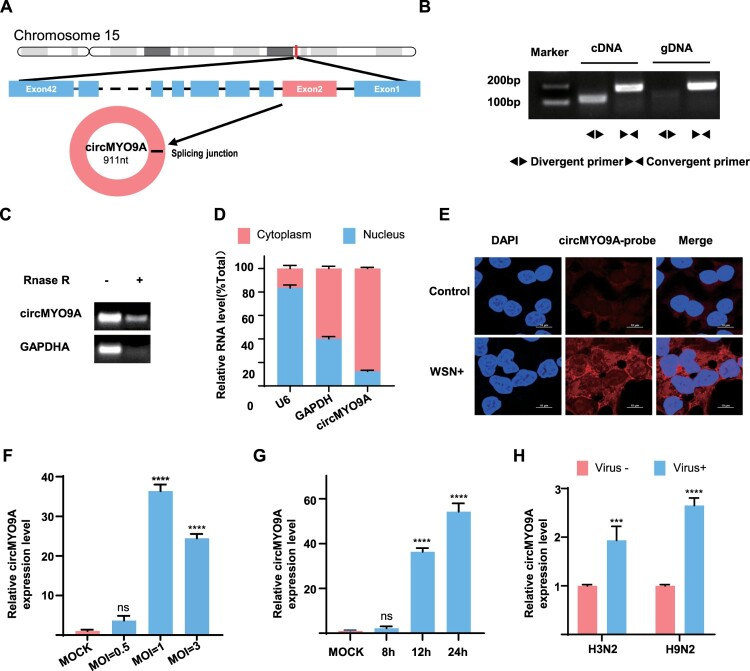
Validation and Characterization of circMYO9A: (A) Genomic loci of the MYO9A gene and circMYO9A. Black arrow indicates the back-splicing of MYO9A exon 2 confirmed by Sanger sequencing. (B) RT-PCR analysis of the existence of circMYO9A with the divergent primers and convergent primers in complementary DNA (cDNA) and genomic DNA (gDNA). (C) RT-PCR analysis of circMYO9A and MYO9A linear mRNA treated with or without RNase R. (D) RT-qPCR analysis of circ MYO9A location in the nucleus or cytoplasm in A549 cells. GADPH served as a marker of cytoplasmic location, while U6 served as a marker of nuclear location, *n* = 3. (E) A549 cells were fixed and subjected to RNA FISH with a biotin-labelled circMYO9A junction specific antisense probe (Red). Nuclei were stained with DAPI (blue). Scale bar, 10 μm. (F) Relative expression of circMYO9A in A549 cells at different MOI. A549 cells were infected with WSN for 12 h, determined by RT-qPCR. (G) Relative expression of circ MYO9A in A549 cells at different time. A549 cells were infected with WSN at an MOI of 1, determined by RT-qPCR. (H) Relative expression of circ MYO9A in A549 cells infected with different subtype IAVs. A549 cells were infected with A/Hong Kong/1/68(H3N2) or A/chicken/Anhui/LH99/2017(H9N2) at an MOI of 1 for 24 h, determined by RT-qPCR. Data shown are the mean ± SD of three samples.

Next, we examined the exon structure of circMYO9A (hsa_circ_0006509), which originates from exon 2 of the MYO9A gene and is 911 nucleotides in length (Figure 2(A)). The back-spliced junction of circMYO9A was amplified via divergent primers and confirmed via Sanger sequencing (Figure 1(F)). To validate the head-to-tail splicing of circMYO9A, convergent and divergent primers specific for the linear or circular form of MYO9A were designed for reverse-transcribed RNA (cDNA) and genomic DNA (gDNA) PCR amplification. RT–PCR analysis of cDNA and gDNA revealed that divergent primers could amplify products from cDNA but not from gDNA (Figure 2(B)). The full sequence of circMYO9A aligns with the circBase database annotation (http://www.circbase.org/). Stability is considered one of the most crucial characteristics of circRNAs. To assess this, RNase R was used to test the resistance of circMYO9A to exonuclease digestion. Total RNA extracted from A549 cells was digested with RNase R exonuclease and subjected to RT–PCR followed by agarose gel electrophoresis. The results revealed that circMYO9A resisted RNase R digestion, unlike the linear GAPDH control (Figure 2(C)). The subcellular localization of circMYO9A was assessed via cytoplasmic and nuclear RNA separation assays and RNA fluorescence in situ hybridization (RNA-FISH). The results demonstrated that circMYO9A was predominantly localized in the cytoplasm (Figure 2(D,E)), suggesting that it may function within the cytoplasm.

Furthermore, we analysed circMYO9A expression in A549 cells infected with various MOI or different viral subtypes and at different time points post-infection (Figure 2(F, G, H)). As shown in Figure 2(F), at 12 h post-infection, the circMYO9A level increased in a dose-dependent manner at MOI ranging from 0.5–1. At an MOI of 3, the circMYO9A level slightly decreased compared with that at an MOI of 1, possibly due to reduced cell numbers following infection at high MOI. Time-course analysis of circMYO9A expression in WSN-infected A549 cells revealed a significant increase in expression as the infection time progressed (Figure 2(G)). As shown in Figure 2(H), different IAVs infections can induce the expression of circMYO9A.

### CircMYO9A is involved in host innate immunity and inhibits viral replication

**Figure 3.**
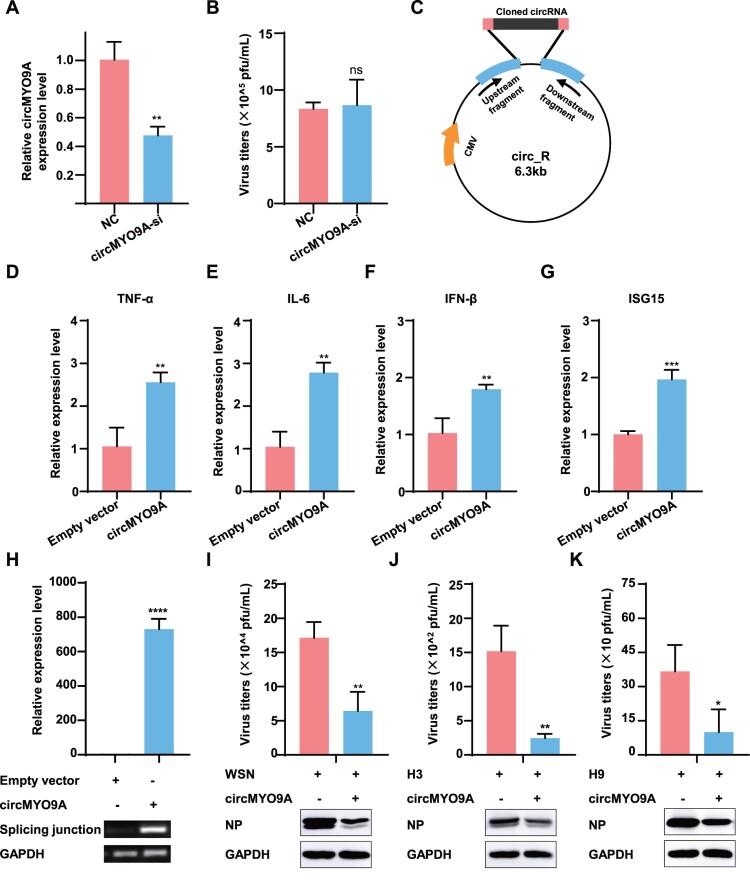
circMYO9A is involved in host innate immunity and inhibits viral replication. (A) siRNAs targeting circMYO9A reduced the expression of circMYO9A. A549 cells were transfected with circMYO9A siRNAs or control siRNA (si-NC), and the relative circMYO9A expression level were determined by RT-qPCR and normalized by the values measured in the control RNA-transfected group. GAPDH was used as internal reference for normalization. Data shown are the mean ± SD of three samples. (B) siRNA-mediated knockdown of circMYO9A did not significantly affect the replication of WSN. A549cells transfected with siRNAs were challenged with WSN (MOI = 0.1). Control siRNA (si-NC) was included as negative control. At 24 hpi, the supernatants of infected cells were collected for plaque assay to determine virus titre. (C) Schematic illustration of the circMYO9A overexpressing plasmid (pcDNA-circMYO9A) construction. (D–G) circMYO9A promotes antiviral innate immunity. RT-qPCR assays were performed to determine the expression levels of TNF-α, IL-6, IFN-β and ISG15 in A549 transfected with circRNA overexpression plasmid (circMYO9A) or Empty vector (EV). (H) Levels of circMYO9A in A549 cells. A549 cells were transfected with pcDNA-circMYO9A and Empty vector, respectively, and the levels of circMYO9A were determined by RT-qPCR 24 h post transfection. RT-qPCR and RT-PCR confirmed A549 stably overexpressed circMYO9A. (I–K) CircMYO9A inhibits different IAVs replication. A549 cells were transfected with pcDNA-circMYO9A (circMYO9A) and Empty vector (EV) respectively, and 24 h later, the cells were infected with different IAV viruses at MOI of 0.1. The supernatants of infected cells were collected 24 h postinfection for plaque assay to determine virus titre. Cell lysates were collected and used for Western blot with anti-IAV NP protein monoclonal antibody.

To explore the biological functions of circMYO9A, small interfering RNAs (siRNAs) targeting circMYO9A and an overexpression plasmid were constructed. Owing to the unique sequence characteristics of the circRNA splicing site, only one siRNA targeting the junction site of circMYO9A was designed. As shown in Figure 3(A), siRNA-mediated knockdown of circMYO9A decreased circMYO9A expression, but silencing circMYO9A did not significantly affect viral replication (Figure 3(B)). Given the low abundance of circMYO9A in uninfected A549 cells and its significant upregulation after viral infection, we constructed a plasmid to overexpress circMYO9A to explore its function further. The pcDNA-circMYO9A plasmid was successfully constructed and significantly increased circMYO9A levels compared with those in virus-stimulated A549 cells (Figure 3(C and H)). Since circRNAs are known to trigger immune responses [13], we focused on the role of circMYO9A in regulating the expression of interferons (IFNs), ISGs, and inflammatory cytokines. As shown in Figure 3(D–G), the overexpression of circMYO9A significantly increased the expression levels of the inflammatory cytokines TNF-α, IL-6, and IFN-β and the antiviral gene ISG15 after WSN infection. We subsequently assessed the effect of circMYO9A on IAV replication to understand its biological importance in the host response. The results revealed that circMYO9A overexpression significantly inhibited the replication of various IAV subtypes 24 h after infection (Figure 3(I, J and K)). In summary, circMYO9A acts as a positive regulator of antiviral immunity, upregulating the expression of inflammatory cytokines and antiviral genes. The upregulation of circMYO9A in virus-infected cells is a host antiviral strategy against IAV infection.

### CircMYO9A does not encode a protein

**Figure 4.**
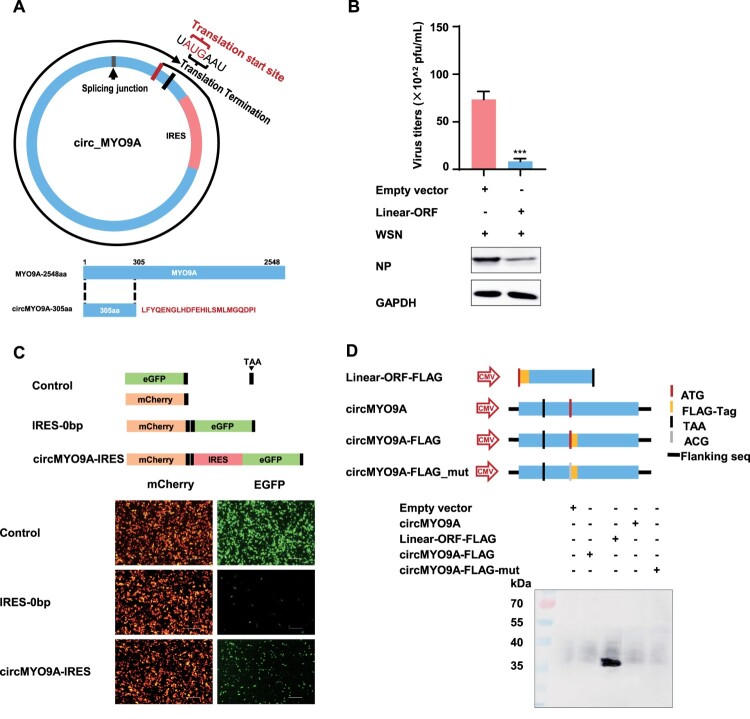
circMYO9A does not encode a protein. (A) Upper panel, the putative open reading frame (ORF) in circMYO9A. Lower panel, the sequences of the putative ORF encoded amino acid sequences are shown. (B) The putative ORF inhibits the replication of IAV in A549 cells. A549 cells were transfected with pcDNA-linear-305aa-orf and Empty vector (EV) respectively, and 24 h later, the cells were infected with WSN at MOI of 0.01. The supernatants of infected cells were collected 24 h postinfection for plaque assay to determine virus titre. Cell lysates were collected and used for Western blot with anti-IAV NP protein monoclonal antibody. (C) The putative IRES activity in circMYO9A was tested. Upper panel, IRES sequences in circMYO9A were cloned between mCherry and EGFP genes with independent start and stop codons as indicated to construct several reporter plasmids. Lower panel, these plasmids were transfected into 293T cells as indicated, IF was performed to determine mCherry and GFP signals. Scale bars, 500 μm. (D) circMYO9A does not translate potential open reading frames. Upper, Schematic diagram of circMYO9A -Flag and Mut plasmid construction. Lower, detection of the expression of nascent protein encoded by circMYO9A overexpressing vector with Flag tag (circMYO9A -Flag) by Western blot with Flag antibody.

Since circRNAs are devoid of cap structure, translation should occur through sequences able to act as internal ribosome entry sites (IRESs). Notably, bioinformatic analysis via IRES finder identified an IRES in circMYO9A, suggesting its potential to encode a protein. The cORF pipeline script was used to detect potential ORFs spanning the junction of the circRNA. A potential ORF in circMYO9A encoding a 305-amino acid protein (referred to as circMYO9A-305aa), which contains a novel sequence with 24 specific amino acids, was identified (Figure 4(A)). To investigate whether the putative ORF possesses antiviral activity, the full-length ORF of circMYO9A was cloned and inserted into the pcDNA3.1 vector. As shown in Figure 4(B), overexpression of the linear circMYO9A ORF significantly inhibited viral replication at 24 h post-infection. While the linear ORF of circMYO9A inhibits IAV replication, further studies are needed to confirm that the 305aa peptides can be translated by circMYO9A. To verify the presence of a natural IRES in circMYO9A, which is required for cap-independent translation initiation, we conducted an IRES activity assay. As shown in Figure 4(C), both mCherry and EGFP were expressed with the putative IRES (173 bp, spanning nucleotides 251 to 424 of circMYO9A), whereas EGFP expression was absent in the absence of the IRES, indicating that the IRES sequence in circMYO9A facilitates ribosome entry and translation initiation. To confirm the presence of circMYO9A-305aa, we constructed three flag-tagged vectors: Linear-ORF-FLAG (FLAG-tagged linear circMYO9A ORF), circMYO9A-FLAG (FLAG-tagged circMYO9A cloned and inserted into the pcDNA3.1 backbone as mentioned above), and circMYO9A-FLAG-mut (FLAG-tagged circMYO9A with a mutated start codon, ATG→ACG) (Figure 4(D)). However, following transfection, the FLAG-tagged circMYO9A-305aa protein was detected at approximately 35 kDa only in the linear ORF group via anti-Flag antibodies. These results collectively demonstrated that the IRES in circMYO9A mediate ribosome entry and can initiate translation. While the putative ORF inhibits IAV replication, circMYO9A does not encode a protein that exerts antiviral effects.

### CircMYO9A is able to regulate the expression and activity of multiple miRNAs

**Figure 5.**
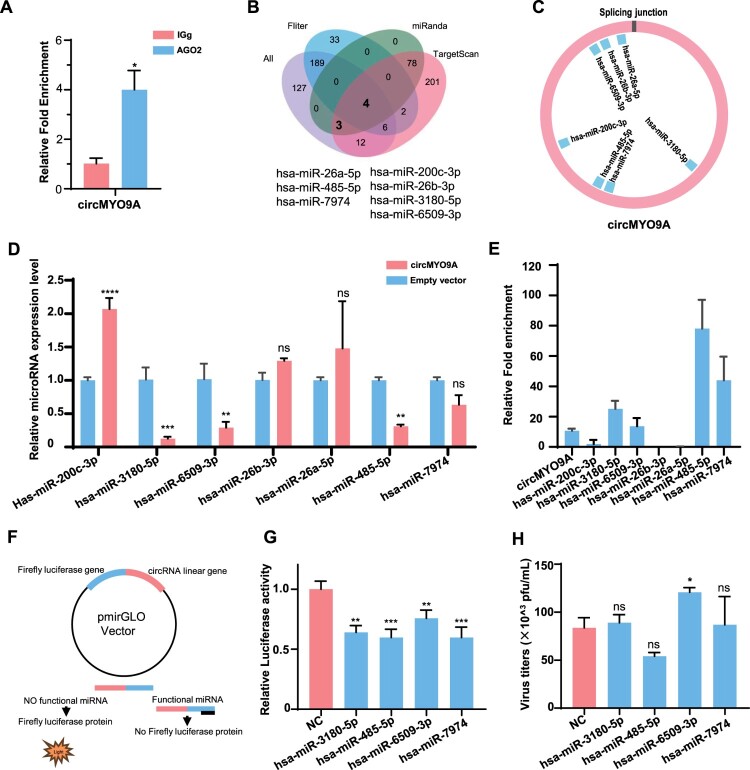
circMYO9A is able to regulate the expression and activity of multiple miRNAs. (A) The Ago2-RIP assay for the amount of circMYO9A in A549 cells transfected Ago2-flag or pcDNA3.1-flag. (B) A schematic illustration showing overlapping of the target miRNAs of circMYO9A predicted by TargetScan, miRanda, and microRNA-seq data. The purple represents the whole detected microRNA, the blue represents the dysregulated microRNA. (C) Schematic drawing showing the putative binding sites of the miRNAs associated with circMYO9A. (D) Relative expression of candidate miRNAs in A549 cells transfected with pcDNA-circMYO9A. (E) CircMYO9A absorbed miRNAs in A549 cells. The relative circMYO9A and miRNA levels pulled down by the probes were determined by RT-qPCR; data shown are values for the circMYO9A-specific probe normalized to those for the control probe. Only the four miRNAs that were enriched (10 to 80-fold higher in the samples assessed by using the circMYO9A-specific probe than in those assessed with the control probe) are shown in the figure. (F) Schematic illustration of the mechanism of action of the pmirGLO vector. (G) The relative luciferase activity was analysed in A549 cells co-transfected with mimics, circMYO9A overexpression plasmid, and control vector. All data represented the mean ± SD from three independent triplicated experiments. ns represent *P* > 0.05; *P < 0.05; **P < 0.01; ***P < 0.001. (H) Effect of four miRNA on influenza virus replication. A549 cells were transfected with miRNA or NC-miR, and 24 h later, the cells were infected with WSN at MOI 0.01. Twenty-four hours after infection, the supernatants were harvested for virus titre determination in MDCK cells. ns represent *P* > 0.05; *P < 0.05; **P < 0.01; ***P < 0.001.

Since circMYO9A is localized primarily in the cytoplasm, we further investigated its potential role as a miRNA sponge. To this end, RNA immunoprecipitation (RIP) assays were performed to pull down RNA transcripts bound to Argonaute-2 (Ago2). After A549 cells were transfected with Ago2-Flag or pcDNA3.1-Flag, RIP assays revealed that circMYO9A could be pulled down by Ago2-Flag (Figure 5(A)), suggesting that circMYO9A might have a binding site for miRNA. To identify miRNAs that interact with circMYO9A, we used miRNA target prediction tools such as TargetScan and miRanda and selected seven candidate miRNAs on the basis of overlap with the miRNA sequencing data (Figure 5(B and C)) (all: the whole detected miRNAs in the miRNA-seq data; filter: the dysregulated miRNAs in the miRNA-seq data). The expression levels of these miRNAs were compared in A549 cells transfected with an overexpression plasmid (circMYO9A) or an empty vector (EV). Among the seven candidate miRNAs, hsa-miR-3180-5p, hsa-miR-6509-3p, hsa-miR-7974 and hsa-miR-485-5p were significantly reduced upon overexpression of circMYO9A (Figure 5(D)). Next, to confirm the direct interaction between circMYO9A and these miRNAs, an RNA pulldown assay with a biotin-labelled circMYO9A probe was performed. The circMYO9A probe, which is specifically antisense to the spliced junction sequence, was conjugated with biotin to enrich both circMYO9A and its associated miRNAs. The results (Figure 5(E)) confirmed that hsa-miR-3180-5p, hsa-miR-6509-3p, hsa-miR-7974 and hsa-miR-485-5p could be sponged by circMYO9A. To exclude the influence of non-specific factors and to assess the ability of these miRNAs to bind to circMYO9A, we subsequently cloned the linear sequence of circMYO9A into the pmirGLO luciferase reporter vector and transfected it with control mimics, hsa-miR-3180-5p, hsa-miR-6509-3p, hsa-miR-7974 and hsa-miR-485-5p mimics into 293T cells (Figure 5(F)). Luciferase assays revealed that compared with NC-miR-transfected cells, cells transfected with these miRNAs presented significantly reduced luciferase activity (Figure 5(G)). To investigate whether these four miRNAs affect IAV infection, we transfected A549 cells with each miRNA or an NC-miR, followed by WSN infection at a multiplicity of infection (MOI) of 0.01. The results showed that hsa-miR-6509-3p mimics facilitate IAV replication, while the viral titres in the other three miRNA-transfected cell lines were similar to those in the NC-miR-transfected cells. These findings suggest that circMYO9A is able to regulate the expression and activity of these four miRNAs and that circMYO9A might function as a sponge of hsa-miR-6509-3p to inhibit viral replication.

### CircMYO9A acts as a sponge for miR-6509-3p to increase SERPINE1 expression

**Figure 6.**
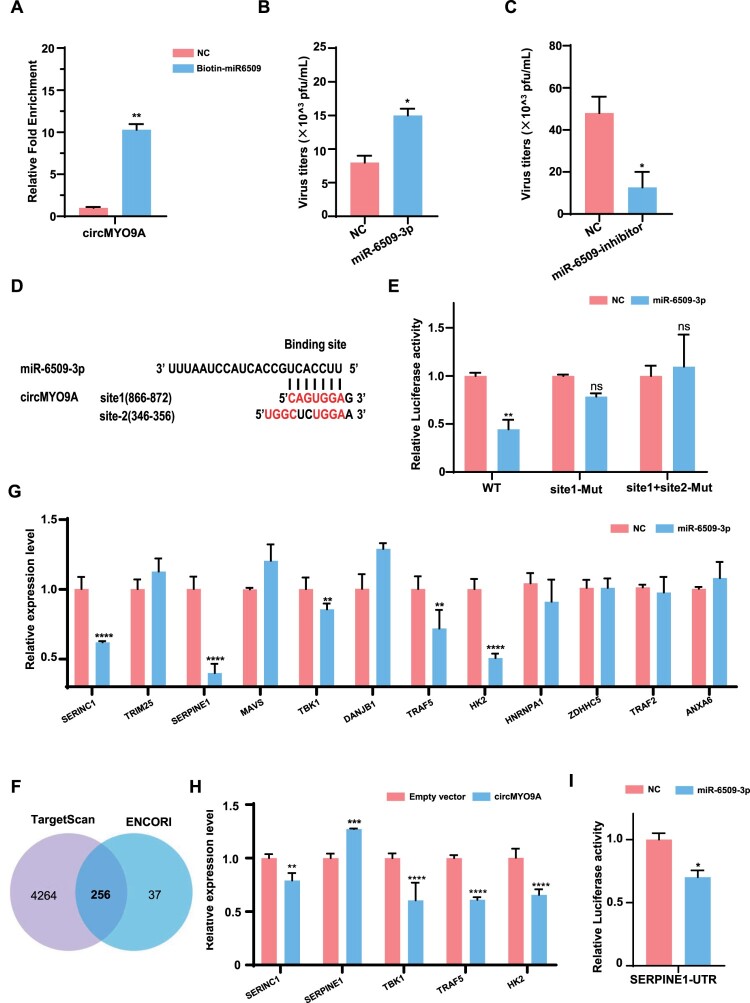
circMYO9A acts as a sponge for miR-6509-3p to increase SERPINE1 expression. (A) RNA pull-down assay was executed in A549 cells, followed by RT-qPCR to detect the enrichment of circMYO9A. (B and C) Effect of miRNA miR-6509-3p and miR-6509-3p inhibitor on influenza virus replication. A549 cells were transfected with miRNA or NC-miR (H) or miRNA inhibitor or negative-control inhibitor (NC inhibitor), and 24 h later, the cells were infected with WSN at MOI 0.01. Twenty-four hours after infection, the supernatants were harvested for virus titre determination in MDCK cells. (D) Predicted binding site of miR-6509-3p in circMYO9A. The predicted binding site sequences in circMYO9A were mutated in the plasmid pmirGLO-circMYO9A as indicated in red. (E) circMYO9A contains two miR-6509-3p binding sites. HEK293T cells were transfected with miR-6509-3p or NC-miR together with pmirGLO-circMYO9A-wt or pmirGLO-circMYO9A-mut. At 36 h post-transfection, a luciferase reporter gene assay was performed to measure luciferase activity. (F) Schematic illustration exhibiting overlapping of the target genes of miR-6509-3p predicted by TargetScan and ENCORI. (G and H) Relative mRNA levels of the target genes of miR-6509-3p were evaluated by RT-qPCR in A549 cells transfected with the miR-6509-3p mimics or circMYO9A plasmid, respectively. (I) MiR-6509-3p affects the luciferase activity of pmirGLO-SERPINE1-UTR.

To further validate the direct interaction between circMYO9A and miR-6509-3p, we performed RNA pulldown assays using biotin-labelled miR-6509-3p. The RT-qPCR results confirmed that circMYO9A was pulled down by biotin-labelled miR-6509-3p but not by the negative control (NC-miR) (Figure 6(A)). A549 cells were subsequently transfected with miR-6509-3p or NC-miR, as well as with miR-6509-3p inhibitors or a control inhibitor (NC inhibitor), and then infected with WSN at an MOI of 0.01. As shown in Figure 6(B and C), the viral titres significantly increased in the miR-6509-3p-transfected group compared with those in the NC group. Conversely, viral titres were lower in the miR-6509-3p inhibitor-transfected group than in the NC inhibitor group. To determine whether the predicted miR-6509-3p binding site in circMYO9A was essential for its interaction, we constructed wild-type and mutated versions of the circMYO9A sequence (Figure 6(D)) and inserted them into the pmirGLO luciferase reporter vector. Luciferase assays revealed a decrease in activity in 293T cells transfected with the wild-type circMYO9A, but this reduction was not fully reversed after mutation at site 1. Furthermore, there was no significant difference between the site 1 and site 2 double mutants and the control, and this reduction was fully restored (Figure 6(E)). These findings suggest that circMYO9A contains two miR-6509-3p binding sites.

Given the limited studies on miR-6509-3p in A549 cells, we sought to explore its role in IAV infection. We hypothesized that circMYO9A inhibits IAV replication by sponging miRNAs. MiRNAs regulate the posttranscriptional expression of target mRNAs by binding to their 3’UTRs. Using TargetScan and ENCORI, we predicted potential target genes of miR-6509-3p (Figure 6(F)). We performed functional annotation and pathway enrichment on the overlapping results of TargetScan and ENCORI, and combined with the GeneCards database, screened out 12 candidate genes involved in antiviral response or innate immunity for further validated (Table.S3). We compared the mRNA levels of these candidate mRNAs in A549 cells transfected with miR-6509-3p or NC, as well as in cells transfected with the circMYO9A overexpression construct and empty vector. As shown in Figure 6(G), compared with NC-miR-transfected cells, miR-6509-3p mimics significantly reduced the expression of the SERINC1, SERPINE1, TBK1, TRAF5 and HK2 genes. Since circMYO9A interacts with miR-6509-3p, it may regulate downstream gene expression by sponging miR-6509-3p. Therefore, we tested whether circMYO9A could modulate the expression of these five candidate genes. As shown in Figure 6(H), only the SERPINE1 mRNA level was upregulated upon circMYO9A overexpression, indicating that circMYO9A might regulate SERPINE1 expression through miR-6509-3p. Thus, SERPINE1 was selected as a target gene of miR-6509-3p for further investigation. To confirm the results of the bioinformatics prediction analysis, the SERPINE1 3’UTR sequence was cloned and inserted into the pmirGLO luciferase reporter plasmid. As shown in Figure 6(I), miR-6509-3p suppressed luciferase activity in the wild-type SERPINE1 3’UTR plasmid, confirming direct binding between miR-6509-3p and the SERPINE1 3’UTR sequence. Collectively, these results demonstrated that circMYO9A directly binds to miR-6509-3p and that miR-6509-3p is able to directly bind to the SERPINE13’UTR to regulate its expression and activity, while circMYO9A modulates SERPINE1 expression via miR-6509-3p. circMYO9A regulates IAV replication by acting as a sponge for miR-6509-3p.

### The circMYO9A-miR-6509-3p-SERPINE1 axis regulates IAV replication

**Figure 7.**
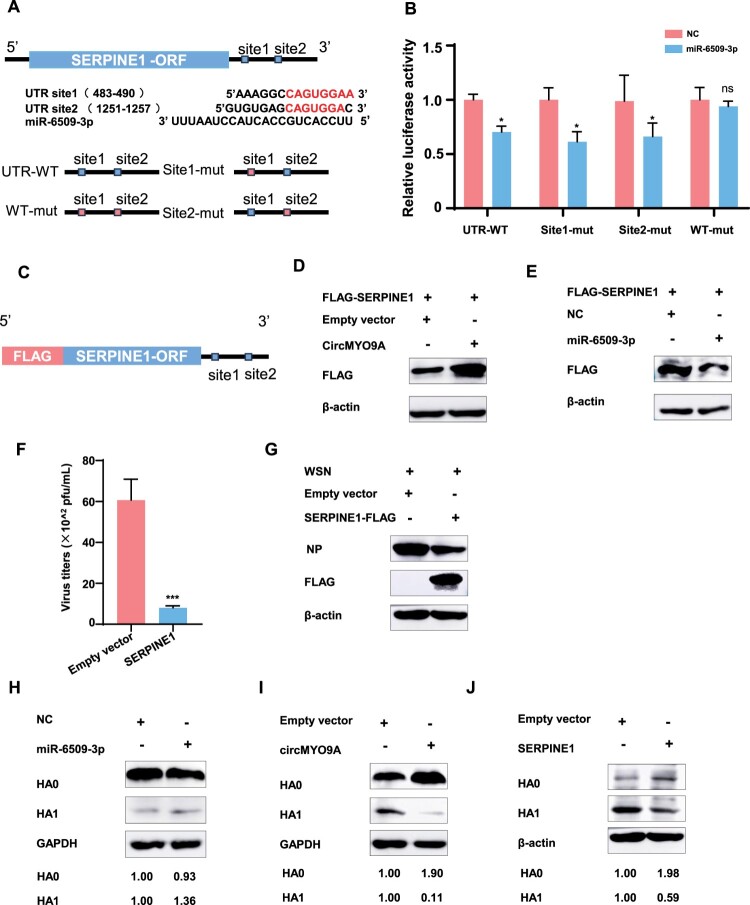
The circMYO9A-miR-6509-3p-SERPINE1 axis regulates IAV replication. (A) Schematic illustration of Predicted binding site of miR-6509-3p in SERPINE1 UTR and the SERPINE1-UTR-wt and SERPINE1-UTR-mut plasmid construction. (B) MiR-6509-3p regulating the luciferase activity of SERPINE1-3'UTR. HEK293T cells were transfected with miR-6509-3p or NC-miR together with pmirGLO-SERPINE1-UTR-wt or pmirGLO-SERPINE1-UTR -mut. At 36 h post-transfection, a luciferase reporter gene assay was performed to measure luciferase activity. (C) Schematic illustration of pCMV-SERPINE1-FLAG plasmid including 3'UTR construction. (D and E) CircMYO9A and miR-6509-3p regulate SERPINE1 protein levels. Protein levels of SERPINE1 were evaluated by western blot in 293T cells co-transfected pCMV-SERPINE1-FLAG together with the miR-6509-3p mimics or circMYO9A plasmid respectively. (F and G) SERPINE1 inhibits the replication of WSN in A549 cells. A549 cells were transfected with PCAGGS-SERPINE1 and Empty vector (EV) respectively, and 24 h later, the cells were infected with different IAV virus at MOI of 0.1. The supernatants of infected cells were collected 24 h postinfection for plaque assay to determine virus titre. Cell lysates were collected and used for Western blot with anti-IAV NP protein monoclonal antibody. (H–J) HA cleavage assay on A549 Cells infected with IAV WSN/33. A549 cells were transfected with NC and miR-6509-3p, or Empty vector (EV) and circMYO9A plasmid, or EV and PCAGGS-SERPINE1-FLAG respectively, and 24 h later, the cells were infected with IAV WSN/33 at MOI of 0.1. Cell lysates were collected and used for Western blot with anti-IAV HA1 protein monoclonal antibody. HA cleavage products HA0 and HA1 are indicated, and relative band intensities are shown at the bottom of the gel.

The SERPINE1 3’UTR sequence was subsequently analysed, and two complementary binding sites for miR-6509-3p were identified (Figure 7(A)). Both the wild-type and mutant forms of the SERPINE1 3’UTR were cloned and inserted into the pmirGLO luciferase reporter vector and co-transfected with miR-6509-3p mimics or control mimics. Luciferase assays revealed that the miR-6509-3p mimic significantly inhibited luciferase activity when the cells were transfected with the wild-type 3’UTR or a single-site mutant 3’UTR, whereas the dual mutant (with both binding sites mutated) did not respond to the miR-6509-3p mimic (Figure 7(B)). Given that circMYO9A interacts with miR-6509-3p and that miR-6509-3p regulates SERPINE1 mRNA expression, we tested whether circMYO9A and miR-6509-3p could influence SERPINE1 protein levels. The SERPINE1 CDS containing the 3’UTR was cloned and inserted into the PCMV-Flag vector (Figure 7(C)), which was subsequently co-transfected with circMYO9A or miR-6509-3p mimics. Western blot analysis revealed that, compared with the control, miR-6509-3p decreased SERPINE1 protein levels, whereas circMYO9A overexpression increased these levels (Figure 7(D and E)). Collectively, these results indicate that miR-6509-3p is able to bind to the SERPINE1 3'UTR directly. MiR-6509-3p or circMYO9A can regulate SERPINE1 protein levels, which is consistent with its role in regulating SERPINE1 mRNA levels, and circMYO9A can regulate SERPINE1 protein levels via miR-6509-3p.

SERPINE1, also known as plasminogen activator inhibitor-1 (PAI-1), is a member of the serine protease inhibitor (SERPIN) family that acts as the primary inhibitor of two main mammalian plasminogen activators, urinary-type (uPA) and tissue-type (tPA). SERPINE1/PAI-1 has also been shown to block surface glycoprotein maturation of influenza A virus, thus reducing virus spread in the airways [34]. We hypothesized that the overexpression of SERPINE1/PAI-1 might restrict viral replication. In A549 cells overexpressing SERPINE1/PAI-1, viral titres and NP protein levels were significantly lower in WSN-infected cells than in control cells at 24 h post-infection (Figure 7(F,G)). SERPINE1/PAI-1 overexpression reduced the specific infectivity of IAV WSN/33 in the presence of trypsin. As IAV does not encode its protease, HA cleavage depends on host proteases during IAV replication [35]. Several proteases known to cleave HA, including urokinase plasminogen activator (uPA; known to be a target of PAI-1), trypsin, human airway trypsin, and tryptase, are inhibited by PAI-1, indicating that PAI-1 can regulate HA cleavage [36]. We examined the influence of SERPINE1/PAI-1 overexpression on HA cleavage in WSN infection in the presence of trypsin. After 24 h of viral infection, overexpression of SERPINE1/PAI-1 blocked trypsin-mediated cleavage of HA0 into HA1 and HA2 (Figure 7(J)), suggesting that SERPINE1/PAI-1 regulates HA maturation and reduces viral infectivity, thereby inhibiting viral replication. Finally, the influence of circMYO9A or miR-6509-3p overexpression on HA cleavage in WSN infection was investigated (Figure 7(H,I)). The overexpression of circMYO9A dramatically reduced HA cleavage, whereas the expression of miR-6509-3p slightly increased HA cleavage during IAV WSN/33 infection. These data collectively demonstrated that circMYO9A acts as a ceRNA for miR-6509-3p, regulating SERPINE1/PAI-1 expression and blocking IAV surface glycoprotein maturation, ultimately reducing viral infectivity and titres. The circMYO9A-miR-6509-3p-SERPINE1 axis plays a crucial role in regulating IAV replication.

### CircMYO9A restricts viral replication and attenuates pathogenicity in a mouse model

**Figure 8.**
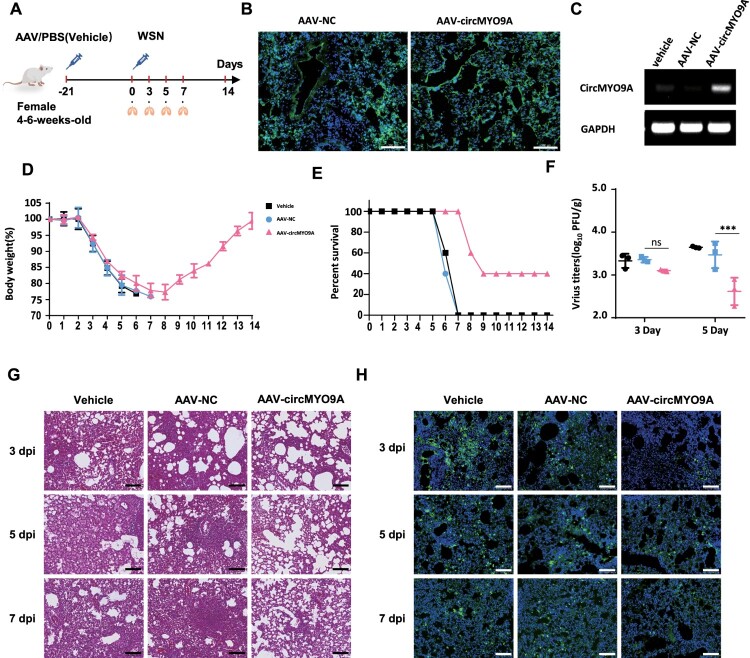
circMYO9A restricts viral replication and attenuates pathogenicity in a mouse model. (A) Experimental design. An adeno-associated virus (serotype 6, AAV6) carrying circMYO9A (AAV6-circMYO9A) or EV (AAV6-NC) was generated. BALB/c (4–6 weeks old, female) mice were administered PBS, AAV6-NC, or AAV6-circMYO9A in a volume of 50 μL (5 × 10^^10^ vector genomes) through intratracheal instil. Then the mice were infected with WSN (5 × 10^^3^ PFU/each) by nasal drip. (B) GFP fluorescent signals in the lung tissue of the AAV-6 injected mice. The lungs of the mice were harvested at 3 weeks post infection. (C) Expression of circMYO9A in mouse lungs. The lungs of the mice were harvested at 3 weeks post infection for RT-PCR analysis of exogenous circMYO9A expression D and E. The body weight and survival rate of the mice were monitored daily for 14 days (*n* = 5). F. The virus titres at 3 and 5 dpi, the lungs of the infected mice (*n* = 3) were harvested for plaque assays (*n* = 3) to measure the viral titres. (G) Pathological changes and viral loading in infected mice lungs. The lungs of infected mice were sliced and the pathological changes of the lungs were assessed by haematoxylin and eosin (H&E) and immunofluorescent staining (IF), NP protein was detected using a mouse anti-NP monoclonal antibody. Scale bars, 100μm.

To investigate the effect of circMYO9A on IAV infection in a mouse model, AAV6-expressing circMYO9A (AAV6-circMYO9A) or negative control AAV6 (AAV6-NC) was delivered into the lungs of BALB/c mice via intratracheal intubation, with PBS used as a control (Figure 8(A)). After treatment with AAV6 for 3 weeks, an EGFP signal was present in the lung tissue, indicating that AAV6 was successfully delivered into the lungs of the BALB/c mice (Figure 8(B)). RT–PCR confirmed that exogenous circMYO9A was expressed in the lungs of the mice at 3 weeks post-infection (Figure 8(C)). We intranasally infected BALB/c mice with WSN and measured their body weights and daily survival. Compared with those treated with AAV6-NC or PBS, the mice treated with AAV6-circMYO9A lost less body weight (Figure 8(D)). Additionally, the mortality rate of the AAV6-circMYO9A-treated mice was lower than that of the AAV-NC- or PBS-treated mice. The AAV6-NC- and PBS-treated mice died within 7 dpi, whereas 40% of the AAV6-circMYO9A-treated mice were still alive at the end of the experiment (Figure 8(E)). The effect of circMYO9A on viral pathogenicity in these mice was also assessed. Histological analysis of the lungs revealed that alveolar damage and interstitial inflammatory cell infiltration were less severe in the AAV6-circMYO9A-treated mice than in the AAV6-NC- or PBS-treated mice at 3, 5, and 7 dpi (Figure 8(G)). To explore the viral load in the lungs, the viral titres in the lungs of the mice at 3 and 5 dpi were examined via plaque assays, and immunofluorescence staining of the lungs was performed with anti-NP mAb. At 3 dpi, although the virus titres were not significantly different among the three groups, those of the AAV6-circMYO9A-treated group were slightly lower than those of the other two groups. The viral titres in the lungs of the AAV6-circMYO9A-treated mice were significantly lower than those in the lungs of the AAV-NC- or PBS-treated mice at 5 dpi (Figure 8(F)). Fewer positive signals were distributed in the lung tissues of AAV6-circMYO9A-treated mice than in those of AAV-NC- or PBS-treated mice at 3, 5, and 7 dpi (Figure 8(H)), indicating a lower viral load in the AAV6-circMYO9A-treated mice. These results indicate that circMYO9A can inhibit viral replication and attenuate pathogenicity in a mouse model, thus increasing the survival of mice during IAV infection, which demonstrates that circRNAs play crucial roles in inhibiting IAV replication and provides novel insights into potential therapeutic strategies involving circRNAs.

## Discussion

CircRNAs, a class of stably expressed noncoding RNAs, have garnered increasing attention because of their association with various diseases, including cancer, cardiovascular disease, and autoimmune disease [5]. However, only a limited number of influenza A virus (IAV)-related circRNAs have been well characterized. In this study, we screened the circRNA transcription profiles of WSN-infected cells and identified a total of 1107 dysregulated circRNAs in WSN-infected A549 cells, of which 794 circRNAs were upregulated, and 313 circRNAs were downregulated. Notably, we identified a circRNA, circMYO9A, which plays a critical role in regulating innate antiviral immune responses and inhibiting IAV replication. Mechanistically, circMYO9A inhibits viral replication by sponging miR-6059-3p as a competing endogenous RNA (ceRNA) for SERPINE1/PAI-1 and upregulating SERPINE1 expression, which restricts IAV haemagglutinin cleavage and subsequently reduces the maturation of progeny particles. In addition, circMYO9A significantly inhibited viral replication in a mouse model, potentially enhancing mouse survival during IAV infection. These results demonstrate that circRNAs play crucial roles in inhibiting IAV replication and provide novel insights into potential therapeutic strategies involving circRNAs.

Accumulating evidence indicates that circRNAs play vital roles in regulating IAV infection and its progression through distinct mechanisms [17,28,30,37,38]. However, the functions of many circRNAs in IAV infection remain unexplored, and further investigations are necessary to elucidate their interactions with the host immune system in antiviral responses. Previous studies have demonstrated that the translation of circRNAs can proceed via a cap-independent mechanism that requires an internal ribosome entry site (IRES) or N6-methyladenosine (m6A)-modifying sequences [39]. Zhao et al. identified an 87-amino-acid peptide encoded by the circular form of the long intergenic nonprotein-coding RNA p53-induced transcript (LINC-PINT), which suppresses glioblastoma cell proliferation both in vitro and in vivo, thereby establishing its potential role in glioblastoma tumorigenesis [40]. Zheng et al. discovered a novel circRNA (circMIB2) in lower vertebrate fish that is translated into a 134 amino acid protein (MIB2-134aa) through m6A modification; this circRNA participates in innate immunity and provides a reference for circRNA therapy in the treatment of pathogenic diseases in economic fish [41]. Our bioinformatic analysis via IRES finder revealed that circMYO9A contains a ribosome entry site (IRES), prompting us to investigate whether it encodes a protein that inhibits viral replication. However, our data indicated that while the IRES in circMYO9A facilitates ribosome entry, circMYO9A does not encode a protein that exerts antiviral effects.

The competing endogenous RNA (ceRNA) hypothesis proposes that RNA transcripts, such as mRNAs, long noncoding RNAs (lncRNAs), and circular RNAs (circRNAs), share microRNA (miRNA) response elements, compete for binding to miRNAs, and thereby regulate the expression of each other, constructing a complex posttranscriptional regulatory network [42]. It has been reported that exonic circRNAs are abundant in the cytoplasm and function as miRNA sponges [43,44]. CircMYO9A, an exonic circRNA primarily localized in the cytoplasm, may regulate viral replication by acting as a miRNA sponge. It is widely well-established that miRNAs regulate target gene expression by binding to Argonaute-2 (Ago2), an essential component of the RNA-induced silencing complex (RISC). In this study, we confirmed the interaction between circMYO9A and AGO2 through RNA immunoprecipitation. Additionally, RNA pulldown and dual-luciferase reporter assays revealed interactions between circMYO9A and candidate miRNAs. The results demonstrated that circMYO9A efficiently absorbs four distinct miRNAs in A549 cells, although only one was associated with viral replication. While the interaction between circMYO9A and the other three miRNAs has not been further investigated, it is reasonable to assume that circMYO9A may have multiple biological functions affecting mRNAs associated with the other three miRNAs. Given the roles of miR-6509-3p and circMYO9A in viral replication, miR-6509-3p was verified as a binding target of circMYO9A. In addition, our study revealed that the miRNA miR6509-3p binds to the untranslated region (UTR) of SERPINE1, suppressing its expression; this suppression could be partially prevented by the upregulation of circMYO9A upon influenza virus infection. Our results indicated that circMYO9A modulates SERPINE1 expression via miR-6509-3p, suggesting a mechanistic role for the circMYO9A-miR-6509-3p- SERPINE1 axis in regulating viral replication.

SERPINE1, which encodes plasminogen activator inhibitor 1 (PAI-1), is a 50 kDa glycoprotein and the main physiological inhibitor of urokinase and tissue plasminogen activator (uPA/tPA), both of which are major regulators of the fibrinolytic system [45]. In addition to inhibiting uPA/tPA, PAI-1 also inhibits multiple serine proteases of the chymotrypsin type with varying efficiencies [46]. Notably, SERPINE1/PAI-1 is also a somewhat unconventional interferon-stimulated gene (ISG), as it is constitutively expressed but can be further upregulated by IFN and other cytokines, such as IL-6, IL-1, TGF-β, and TNF-α [47,48]. As shown in Fig.S1, exogenous IFN-β stimulation could upregulate SERPINE1 expression in A549 cells, suggesting that SERPINE1 could function as an ISG. Our results showed that circMYO9A could facilitate the upregulation of inflammatory cytokines (TNF-α, IL-6) and antiviral factors (IFN-β, ISG15) following WSN infection, which, in turn, might further enhance SERPINE1 expression, suggesting a potential synergy between circMYO9A-mediated immune activation and its ceRNA function in regulating SERPINE1. Previous studies have reported that PAI-1 targets host proteases such as human tryptase, HAT, TMPRSS2 and Furin to protect the host during natural IAV infection [34,49]. Haemagglutinin (HA) can be transported to the cell surface and incorporated into virions in an uncleaved form, with cleavage subsequently mediated by soluble proteases such as plasmin in the extracellular space or by unknown serine protease(s) in endosomal vesicles of target cells [50]. Our results suggest that the overexpression of SERPINE1/PAI-1 reduces HA cleavage in WSN infection in the presence of trypsin, further indicating that SERPINE1/PAI-1 may target different proteases at various cellular locations, thereby inhibiting IAV haemagglutinin cleavage and reducing the infectivity of progeny viruses. This effect directly reduces the infectivity of progeny viruses, particularly during multiple rounds of infection. circMYO9A inhibited IAV replication by sponging miR-6059-3p as a competing endogenous RNA (ceRNA) for SERPINE1/PAI-1 and upregulating SERPINE1 expression, which restricted IAV haemagglutinin cleavage and subsequently reduced the maturation of progeny particles. Meanwhile, the immune activation mediated by CircMYO9A also induces the expression of SERPINE1 to a certain extent, suggesting that it may cooperate with the ceRNA function to jointly regulate SERPINE1. Thus, the upregulation of circMYO9A may represent an antiviral strategy for the host following viral infection, which may tip the balance back towards the host. In addition, we further evaluated the effect of circMYO9A on the IAV entry process, including attachment, internalization, membrane fusion, and uncoating (Fig.S4), employing the methodologies described in prior studies [51–53]. The results indicated that circMYO9A has no effect on the step of viral entry. Because circMYO9A does not have a homologous circRNA in mice, we investigated the effect of circMYO9A on IAV infection in a mouse model. Our results indicate that circMYO9A can inhibit viral replication and attenuate pathogenicity in a mouse model, thus potentially increasing the survival of mice during IAV infection. These findings demonstrate that circRNAs play crucial roles in inhibiting IAV replication and provide novel insights into potential therapeutic strategies involving circRNAs.

Our study thus provides new insights into the roles of circRNAs in the cellular innate antiviral response. CircRNAs have attracted significant attention because of their involvement in various biological processes. However, the functions of many circRNAs during viral infection remain unknown, necessitating further investigations to understand their interactions with the host immune system in antiviral defence responses. Exploring the physiological functions of circRNAs will enhance our understanding and offer potential therapeutic strategies for influenza virus infection.

## Supplementary Material

SUPPLEMENTARY_MATERIALS.docx

SupplementaryFigure_3.eps

TableS3.xlsx

SupplementaryFigure_1.eps

TableS2.xlsx

SupplementaryFigure_2.eps

TableS1.docx

graphical_abstract.tif

SupplementaryFigure_4.eps
