# Supplementary material for: CircMYO9A inhibits influenza A virus replication by dampening haemagglutinin cleavage via increasing SERPINE1/PAI-1 expression
Source: Emerg Microbes Infect. 2025 May 2;14(1):2502007. doi: 10.1080/22221751.2025.2502007 (PMC12093801; doi:10.1080/22221751.2025.2502007)
Supplement: SUPPLEMENTARY_MATERIALS.docx [file TEMI_A_2502007_SM1041.docx]

**SUPPLEMENTARY MATERIALS**

**Fig.S1. Exogenous IFN-β stimulation could upregulate SERPINE1 expression in A549 cells.**

A549 cells were treated with 100 ng/mL IFN-β. After 24 h, the expression level of ISG15(A) and SERPINE1(B) was determined by qRT-PCR.

**Fig.S2 The effect of circMYO9A on HSV and VSV replication**

(A) The effect of circMYO9A on HSV replication

(B) The effect of circMYO9A on VSV replication.

A549 cells were transfected with either empty vector or pcDNA-circMYO9A. After 24h of transfection, the cells were infected with HSV (MOI = 0.1) or VSV-GFP (MOI = 0.5). Viral titers in the supernatants were measured 24 hours post-infection. Data are presented as means ± SD. "ns" indicates no statistically significant difference.

**Fig.S3 The effect of circMYO9A on the step of viral entry**

A549 cells transfected with the empty vector, pcDNA-circMYO9A were infected with WSN (MOI=5) for 1 h at 4 °C. At 4 °C condition, the influenza virus can only initiate adsorption but not endocytosis. (A) For the viral attachment assay, cells were washed three times with cold PBS (pH=7.4) to remove unbound virions. Then the cells were lysed and collected, the vRNA levels of viral NP gene and protein expression levels of viral NP were determined using RT-qPCR and western blot respectively. (B) For the viral internalization assay, after the same treatment, cells were washed three times with cold PBS (pH=7.4) to remove the unbound virions. Since acidic agents can undock the virus particles adsorbed to the cell surface, but not those that have been internalized, cells were then incubated in pre-warmed Opti-MEM for 1 h at 37 °C and washed three times with cold PBS (pH=1.5) to remove bound but non-internalized virions. Cell lysates were harvested, the vRNA level of viral NP gene and protein expression level of viral NP were determined in the same manner.

(C)For fusion assays, WSN (H1N1) virus was labelled with R18 (octadecyl rhodamine B chloride) and SPDiOC18[3,3=-dioctadecyl-5,5=-di(4-sulfophenyl) oxacarbocyanine] (Life Technologies) at final concentrations of 23 and 46 μM, respectively. After intense vortexing at RT for 1 h, the labelled viruses were filtered through a 0.22-μm filter. A549 cells seeded in glass-bottom dishes were transfected with the empty vector, pcDNA-circMYO9A for 24 h, and then infected with the labelled WSN(H1N1) virus for 30 min on ice. After being washed with PBS, the cells were shifted to 37°C for 0 or 180 min, fixed with 4% PFA, treated with 0.5% Triton X-100 in PBS, and visualized by confocal microscopy.

(D) For uncoating assays, A549 cells seeded in glass-bottom dishes were transfected with the empty vector, pcDNA-circMYO9A for 24 h, and then infected with WSN (H1N1) virus at an MOI of 10 on ice for 1 h. The cells were washed three times, incubated at 37°C for 3 h in the presence of 1 mM CHX to block viral protein synthesis, and fixed with 4% PFA. The cells were then stained with a rabbit anti-M1 pAb and Alexa Fluor 488 goat anti-rabbit IgG (H + L), and visualized by confocal microscopy.

**Fig.S4**

(A) siRNA-mediated knockdown of circMYO9A did not significantly affect the replication of influenza A/H3N2 and (B) influenza A/H9N2virus. A549cells transfected with siRNAs were challenged with H3 and H9 IAVs (MOI = 0.1), respectively. Scramble siRNA was included as negative control. At 24 hpi, the supernatants of infected cells were collected for plaque assay to determine virus titer. Abbreviation: ns, not significant.

(C) shRNA-mediated knockdown of circMYO9A reduced the expression of circMYO9A. The shRNA sequence targeting circMYO9A was insert into pLKO.1-puro plasmid (MISSION Sigma-Aldrich). A549 cells were transfected with shRNA plasmid or empty vector, and the relative circMYO9A expression level were normalized by the values measured in the control RNA-transfected group. GAPDH was used as internal reference for normalization. Scale bar, 10 μm.

(D)shRNA-mediated knockdown of circMYO9A did not significantly affect the replication of WSN, (E)influenza A/H3N2 and (F) influenza A/H9N2 virus. A549cells transfected with shRNA plasmid were challenged with WSN, H3 and H9 IAVs (MOI = 0.1). empty vector was included as negative control. At 24 hpi, the supernatants of infected cells were collected for plaque assay to determine virus titers. Scale bar, 10 μm.

**Table.S1** Sequences of primers, probes, siRNA, miRNA, and miRNA inhibitors used in this study.

**Table.S2** CircMYO9A-Numerical data

**Table.S3** Candidate gene information
