# Supplementary material for: CircMYO9A inhibits influenza A virus replication by dampening haemagglutinin cleavage via increasing SERPINE1/PAI-1 expression
Source: Emerg Microbes Infect. 2025 May 2;14(1):2502007. doi: 10.1080/22221751.2025.2502007 (PMC12093801; doi:10.1080/22221751.2025.2502007)
Supplement: TableS1.docx [file TEMI_A_2502007_SM1035.docx]

Supplementary Table 1. Sequences of primers, probes, siRNA, miRNA, and miRNA inhibitors used in this study.

| Purpose | Primer | Sequence (5'-3') |
| --- | --- | --- |
| qRT-PCR | GAPDH-F | ACAACTTTGGTATCGTGGAAGG |
|  | GAPDH-R | GCCATCACGCCACAGTTTC |
|  | novel_circ_000346_F | ACTTACCATTTCATCTCCCCTG |
|  | novel_circ_000346_R | CCTTGGTTACACCAACAGTACG |
|  | novel_circ_006872_F | GTTTCTTGCCATCTTACAAGCC |
|  | novel_circ_006872_R | AATCTGCTTGTGTGTGCGT |
|  | novel_circ_010280_F | TGATGCAAAACCTTCAGCC |
|  | novel_circ_010280_R | TGACTTCCACCTGCCTCTCG |
|  | circMYO9A_F | GCTGGACCAGTACTTGAGTTA |
|  | circMYO9A _R | TATCTTCAAAGCGTCGTCTTCC |
|  | has_circ_001713_F | CTACTGCAATGGCCATGCTTG |
|  | has_circ_001713_R | CACTTGTCACAGGTTAGCAC |
|  | has_circ_001589_F | TAATTGTGGTTGCGTGAGAG |
|  | has_circ_001589_R | GGTCTAACAGTGTAACTCTTGTC |
|  | hsa-miR-200c-3p-RT | GTCGTATCCAGTGCAGGGTCCGAGGTATTCGCACTGGATACGACTCCATC |
|  | hsa-miR-26b-3p-RT | GTCGTATCCAGTGCAGGGTCCGAGGTATTCGCACTGGATACGACAGCCAA |
|  | hsa-miR-3180-5p-RT | GTCGTATCCAGTGCAGGGTCCGAGGTATTCGCACTGGATACGACCGACGT |
|  | hsa-miR-6509-3p-RT | GTCGTATCCAGTGCAGGGTCCGAGGTATTCGCACTGGATACGACAAATTA |
|  | hsa-miR-26a-5p-RT | GTCGTATCCAGTGCAGGGTCCGAGGTATTCGCACTGGATACGACAGCCTA |
|  | hsa-miR-485-5p-RT | GTCGTATCCAGTGCAGGGTCCGAGGTATTCGCACTGGATACGACGAATTC |
|  | hsa-miR-7974-RT | GTCGTATCCAGTGCAGGGTCCGAGGTATTCGCACTGGATACGACGGGCTC |
|  | hsa-miR-200c-3p-F | CGCGTAATACTGCCGGGTAAT |
|  | hsa-miR-26b-3p-F | CGCGCCTGTTCTCCATTAC |
|  | hsa-miR-3180-5p-F | CCAGACGCTCCGCCCC |
|  | hsa-miR-6509-3p-F | GCGTTCCACTGCCACTACC |
|  | hsa-miR-26a-5p-F | CGCGTTCAAGTAATCCAGGA |
|  | hsa-miR-485-5p-F | CGAGAGGCTGGCCGTGAT |
|  | hsa-miR-7974-F | CGAGGCTGTGATGCTCTCCT |
|  | hsa-miR-R | CAGTGCAGGGTCCGAGGTAT |
|  | U6-F | CTCGCTTCGGCAGC ACA |
|  | U6-R | AACGCTTCACGAATTTGCGT |
|  | TNF-α-hum-F | GTAGCCCATGTTGTAGCAAACC |
|  | TNF-α-hum-R | GAGGTACAGGCCCTCTGATG |
|  | IFN-β-hum-F | ATGACCAACAAGTGTCTCCTCC |
|  | IFN-β-hum-R | GGAATCCAAGCAAGTTGTAGCTC |
|  | IL-6-hum-F | TTCTCCACAAGCGCCTTCGGTC |
|  | IL-6-hum-R | TCTGTGTGGGGCGGCTACATCT |
|  | ISG15-F | CGCAGATCACCCAGAAGATCG |
|  | ISG15-R | TTCGTCGCATTTGTCCACCA |
|  | SERINC1_F | GATGTTGGTATGCAGCCTTGT |
|  | SERINC1_R | CAACGCAGAGGAGCATGTTG |
|  | SERPINE1-F | AGTGGACTTTTCAGAGGTGGA |
|  | SERPINE2-R | GCCGTTGAAGTAGAGGGCATT |
|  | TBK1_F | TGCACCCTGATATGTATGAGAGA |
|  | TBK1_R | AAATGGCAGTGATCCAGTAGC |
|  | TRAF5_F | CCACTCGGTGCTTCACAAC |
|  | TRAF5_R | GTACCGGCCCAGAATAACCT |
|  | HNRNPA1_F | CAGCTATAACAACGGAGGCGG |
|  | HNRNPA1_R | ACCGCCATAGCCACCTTGG |
|  | ZDHHC5_F | CACCTGCCGCTTTTACCGT |
|  | ZDHHC5_R | CGGCGACCAATACAGTTATTCAC |
|  | TRAF2_F | GCTCATGCTGACCGAATGTC |
|  | TRAF2_R | GCCGTCACAAGTTAAGGGGAA |
|  | ANXA6_F | ACGGTTGATTGTGGGCCTG |
|  | ANXA6_R | GTGCATCTGCTCATTGGTCC |
|  | TRIM25_F | AGCAGCTACAACAAGAATACACG |
|  | TRIM25_R | GGCTCTGTTCAATCTCCTCCT |
| CircMYO9A overexpression plasmid (pcDNA- CircMYO9A) construction | Upstream-F | tagtccagtgtggtggaattcTTTCTTAATCATCTGAAGCATGGAG |
|  | Upstream-R | gccgccactgtgctggatatcTGGACTTAGATTTTGTTCCTAAACAG |
|  | Downstream-F | gcacagtggcggccgctcgagTGGACTTAGATTTTGTTCCTAAACAG |
|  | Downstream-R | ggtttaaacgggccctctagaTTTCTTAATCATCTGAAGCATGGAG |
|  | Linear circMYO9A with flanking sequence-F | caaaatctaagtccagatatcTTGCTGTGCTAAGACAAGAGTTA |
|  | Linear circMYO9A with flanking sequence-R | tctaagtccaGATGGAACAGTATCTTGCTTG |
| CircMYO9A overexpression plasmid with Flag tag (pcDNA- CircMYO9A) construction | CircMYO9A _N_FLAG-F | gattacaaggacgacgatgacaagAATATAAATGATGGAGGAAGACGACG |
|  | CircMYO9A -N-FLAG-R | cttgtcatcgtcgtccttgtaatccatCATATTGGATCCTGTCCCATCAGCATG |
|  | CircMYO9A _N_ATG-Mut-R | cttgtcatcgtcgtccttgtaatccGtCGTATTGGATCCTGTCCCATCAGCATG |
|  | CircMYO9A -orf-N-FLAG-F | AGTGTGGTGGaattcATGGATTACAAGGACGACGATGACAAGAATATAAATGATGGAGG |
|  | CircMYO9A -orf-N-FLAG-R | AGCGGCCGCCACTGTGCTGGATatcTTATATTGGATCCTGTCCCATCAGC |
| CircMYO9A luciferase reporter plasmid (pmirGLO- CircMYO9A -wt) construction | CircMYO9A linear sequence-F | tgtttaaacgagctcgctagcTTATTTTACCAAGAAAATGG |
|  | CircMYO9A linear sequence-R | tgcctgcaggtcgactctagaCTCAAGTACTGGTCC |
| CircMYO9A luciferase reporter plasmid (pmirGLO - CircMYO9A -mt) construction | Mutant overlap primer -R | CTCAAGTACTGGTCCAGCTCCAAGAATAATCTGTTCTAcaggtgacGCAAATCCTTTC |
|  | Mutant overlap primer -site1-F | TCACCATCACCCTAGACTCAAAGCACAAACTTTC |
|  | Mutant overlap primer - site1-R | ctagggtcttggtgaCTCTCCTGAAATCACGATG |
|  | Mutant overlap primer -site2-F | ccccgaaaccgTCaccttAATCGCTTAAGTGGAGAG |
|  | Mutant overlap primer -site2-R | aaggtGAcggtTTCGGGGCCACAGCATCATTC |
| AGO2-Flag expression vector | PCMV-AGO2-F | aaggacgacgatgacaagcttATGTACTCGGGAGCCGGCCCCG |
|  | PCMV-AGO2-R | agatctcggtcgaccgaattcTCAAGCAAAGTACATGGTGCGC |
| SERPINE1-Flag expression vector | PCA-SERPINE1-CDS-F | gacgacgatgacaaggaattcATGGGATTCAAGATTGATGACAAG |
|  | PCA-SERPINE1-CDS-R | aaaaagatctgctagctcgagTCAGGGTTCCATCACTTGGC |
| The SERPINE1 expression reporter vectors | pcmv-SERPINE1-2400-F | aaggacgacgatgacaagcttATGGGATTCAAGATTGATGACAAG |
|  | pcmv-SERPINE1-2400-R | gatctcggtcgaccgaattcCTTGAACTCCTGGGCTCAAGC |
| Serpine1 luciferase reporter plasmid construction (pmirGLO Serpine-UTR site1 and site2) | Serpine-UTR-R | gttgtttaaacgagctcgctagc CACTGAGGCCCTTTGCAGGATGGAAC |
|  | Serpine-UTR-F | tgcctgcaggtcgactctaga CTTGAACTCCTGGGCTCAAGCAATC |
| mutant Serpine1 luciferase reporter plasmid construction | Site1-mut-R | GTTTCAAGGTGACGCCTTTGGCCTGTCACCAGCCTC |
|  | Site1-mut-F | CCAAAGGCGTCACCTTGAAACACCCTTTCATCTCAGAGTCC |
|  | Site2-mut-R | GTGaggtgacCTCACACACAGCAGCCGGAAATGAC |
|  | Site2-mut-F2 | GTGTGAGgtcacctCACGTGAGGGGGGGGTGGGTGAGA |
| siRNA | CircMYO9A-siRNA | sense: AGUACUUGAGUUAUUUUACTT |
|  |  | antisense: GUAAAAUAACUCAAGUACUTT |
|  | si-NC | sense: UUCUCCGAACGUGUCACGUTT |
|  |  | antisense: ACGUGACACGUUCGGAGAATT |
| circMYO9A-shRNA-Sense |  | GATCCGGACCAGTACTTGAGTTATTTCTCGAGAAATAACTCAAGTACTGGTCCTTTTTG |
| circMYO9A-shRNA-Anti-Sense |  | AATTCaaaaaGGACCAGTACTTGAGTTATTTCTCGAGAAATAACTCAAGTACTGGTCCG |
| FISH | CircMYO9A-cy3 probe | AAATAAC+TCAAGTAC+TGGTCCAGCT |
|  | Control-biotin probe | biotin-CTAAGACTAGGTGTTCGGAGGGAAAACAAAAAGAGATATCAGAA |
|  | hsa-miR-6509-3p RNA pulldown Probe | 5'Biotin -UUCCACUGCCACUACCUAAUUU |
| Synthesized miRNAs | hsa-miR-485-5p mimics | sense: AGAGGCUGGCCGUGAUGAAUUC |
|  |  | antisense: AUUCAUCACGGCCAGCCUCUUU |
|  | hsa-miR-3180-5p mimics | sense: CUUCCAGACGCUCCGCCCCACGUCG |
|  |  | antisense: ACGUGGGGCGGAGCGUCUGGAAGUU |
|  | hsa-miR-6509-3p mimics | sense: UUCCACUGCCACUACCUAAUUU |
|  |  | antisense: AUUAGGUAGUGGCAGUGGAAUU |
|  | hsa-miR-7974 mimics | sense: AGGCUGUGAUGCUCUCCUGAGCCC |
|  |  | antisense: GCUCAGGAGAGCAUCACAGCCUUU |
|  | NC-miR | sense: UUCUCCGAACGUGUCACGUTT |
|  |  | antisense: ACGUGACACGUUCGGAGAATT |
| Synthesized miRNA inhibitor | hsa-miR-6509-3p inhibitor | AAAUUAGGUAGUGGCAGUGGAA |
|  | NC inhibitor | UUCUCCGAACGUGUCACGUTT |
